# Activity of Saponins from *Medicago* Species against Phytoparasitic Nematodes

**DOI:** 10.3390/plants9040443

**Published:** 2020-04-02

**Authors:** Trifone D’Addabbo, Maria Pia Argentieri, Jerzy Żuchowski, Elisa Biazzi, Aldo Tava, Wieslaw Oleszek, Pinarosa Avato

**Affiliations:** 1 Institute for Sustainable Plant Protection, National Council of Research, 70125 Bari, Italy; 2 Department of Pharmacy–Drug Sciences, University of Bari Aldo Moro, 70125 Bari, Italy; mariapia.argentieri@uniba.it (M.P.A.); pinarosa.avato@uniba.it (P.A.); 3 Department of Biochemistry, Institute of Soil Science and Plant Cultivation–State Research Institute, 24-100 Pulawi, Poland; jzuchowski@iung.pulawy.pl (J.Ż.); wo@iung.pulawy.pl (W.O.); 4 CREA-Research Centre for Animal Production and Acquaculture, 26900 Lodi, Italy; elisa.biazzi@crea.gov.it (E.B.); aldo.tava@crea.gov.it (A.T.)

**Keywords:** *Medicago*, saponins, *Meloidogyne incognita*, *Xiphinema index*, *Globodera rostochiensis*, sustainable management

## Abstract

Content of bioactive saponins of *Medicago* species suggests that they may also exert, as previously demonstrated on *M. sativa*, nematicidal properties exploitable for the formulation of new products for sustainable phytoparasitic nematode management. This study was addressed to highlight the bioactivity of saponins from five different *Medicago* species still poorly known for their biological efficacy, i.e., *M. heyniana, M. hybrida, M. lupulina*, *M. murex* and *M. truncatula*, against the plant parasitic nematodes *Meloidogyne incognita*, *Xiphinema index* and *Globodera rostochiensis*. The bioactivity of the extracts from the five *Medicago* species was assessed by in vitro assays on the juveniles (*J2*) and eggs of *M. incognita* and *G. rostochiensis* and the adult females of *X. index*. The suppressiveness to *M. incognita* of soil treatments with the *Medicago* plant biomasses was also investigated in a tomato experiment. The nematicidal activity of the five *Medicago* species was reported and discussed in relation to their phytochemical profile.

## 1. Introduction

Plants can be a large source of biocidal compounds potentially suitable to formulate new pesticides for sustainable management of plant pathogens and pests, also including phytoparasitic nematodes [1]. 

Presence of a variety of nematicidal phytochemicals in many botanical families has increasingly focused the attention of scientists and farmers to plant-derived nematicidal products [2,3]. Previous studies of our research group documented a strong activity on phytoparasitic nematodes for chemical constituents of extracts from Asteraceae and Brassicaceae plants, such as phenolics and glucosinolates [4,5], as well as for the essential oils from many aromatic and medicinal plants [6,7].

Saponins represent a wide group of specialized phytochemicals, consisting of a triterpene or steroid aglycone to which one or more sugar chains are attached, present in many plant families but particularly abundant in the Fabaceae plants [8]. Saponins were acknowledged for a wide range of biological activities, including a cytotoxic, antibiotic, anti-inflammatory and molluscicidal activity [8,9]. In addition, a high anthelmintic activity against gastrointestinal nematodes from donkey [10] and goats [11] was also proved for alfalfa (*Medicago sativa* L.) saponin mixtures.

The activity of saponins was also demonstrated on phytoparasitic nematodes, as affecting juvenile (*J2*) motility or egg and *J2* viability of the root-knot nematodes *Meloidogyne incognita* Kofoid et White (Chitw) [12] and *M. javanica* Treub [13]. Moreover, saponin-rich extracts from *Quillaja saponaria* Molina were found to have significant nematicidal effects on other economically relevant phytonematode species, such as the dagger nematode *Xiphinema index* Thorne et Allen and the root lesion nematode *Pratylenchus thornei* Sher et Allen [14], as well as significantly reducing *M. incognita* infestation on field tomato (*Solanum lycopersicum* L.) or melon (*Cucumis melo* L.) crops [15]. 

The *Medicago* genus (Fabaceae family) contains 83 different herbaceous or shrub plant species, mainly distributed around the Mediterranean basin but also adapted to a large range of environmental conditions, which produce several specialized metabolites such as coumarins, flavonoids, naphtoquinones, alkaloids and also saponins [8,16,17,18,19]. 

Saponins from *Medicago* spp. are formed by complex mixtures of high molecular weight triterpene glycosides with medicagenic acid, hederagenin, zahnic acid, bayogenin and soyasapogenols A and B as the dominant aglycones [20,21]. *Medicago* saponins have been reported to possess a large spectrum of biological and pharmacological effects, such as cytotoxic, antitumor, fungicidal, molluscicidal, antibacterial and antiviral activities [8,22,23,24]. 

Previous studies of our research group assessed also the nematotoxic potential of active saponins obtained from various *Medicago* species. Argentieri et al. (2008) [25] described the nematicidal activity of saponins and derived prosapogenins and sapogenins from *M. arborea*, *M. arabica* and *M. sativa* against *X. index*, as well as in vitro bioassays demonstrated the activity of saponin mixtures from *M. sativa* against *X. index*, *M. incognita* and the potato cyst nematode *Globodera rostochiensis* Wollenweber [26]. The species *M. heyniana* Greuter, *M. hybrida* (Pourr.) Trautv., *M. lupulina* L., *M. murex* Willd. and *M. truncatula* Gaertn. are also widespread as fodder plants throughout the Mediterranean basin but, adversely to *M. sativa*, their biological activities were scarcely documented. The saponin content of these species suggests that they may also possess nematicidal properties exploitable for the formulation of new nematicidal products Therefore, a study was carried out to investigate the nematicidal activity of saponin-rich extracts from these five *Medicago* species against the phytoparasitic nematodes *M. incognita*, *X. index* and *G. rostochiensis*, as well as to assess the suppressiveness of soil amendments with their plant material to *M. incognita* on tomato.

## 2. Results

### 2.1. Saponin Content and Composition

Crude saponin content (% of dry matter) of the five *Medicago* species under study is depicted in Table 1. Saponin amount differs in the five species, as ranging from 0.60 ± 0.05% in *M. truncatula* to 1.62 ± 0.30% in *M. heyniana*. The crude saponin mixtures were purified at a highly pure grade (80%–90%) by reverse-phase chromatography as here reported and then used for the chemical and biological studies.

**Figure 1:**
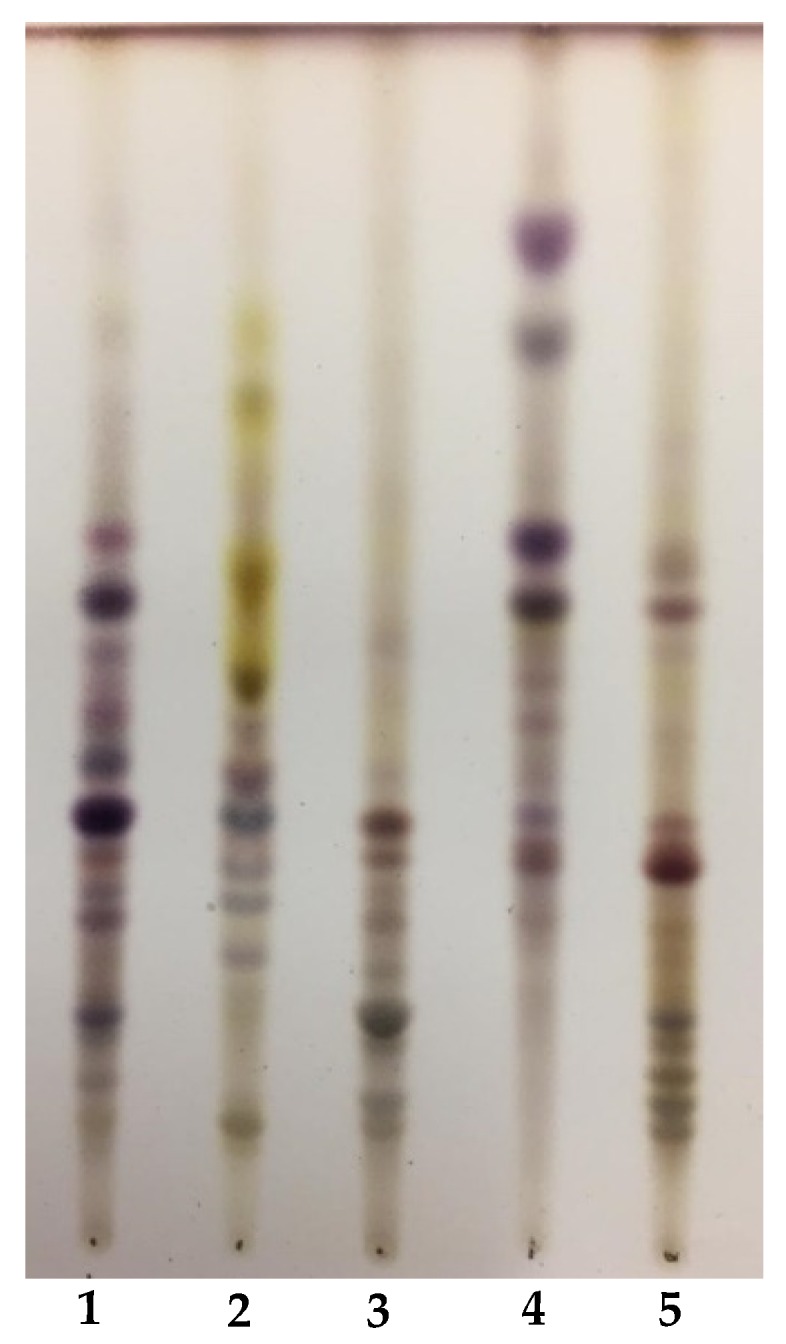
TLC of the five saponin extracts. **1:** *M. heyniana*; **2:** *M. hybrida*; **3:** *M. lupulina*; **4:** *M. murex*; **5:** *M. truncatula*. Spots were visualized by Liebermann–Burckard reagent.

Preliminary investigation by TLC of the crude saponin extracts from the five *Medicago* species (Figure 1) revealed a complex chemical profile rich in several constituents, which overall indicated a quite different composition of the five crude extracts. Since the biological activity of saponins is related to both the aglycone moieties and the saccharidic parts, characterization of both aglycones and sugars has been performed. The evaluation of total aglycone moieties is reported in Table 2, while saponin composition (including preliminary evidence of their glycosidic moieties) is listed in Table 3.

Gas Chromatography/Flame-Ionization Detection (GC/FID) and GC/Mass Spectrometry (GC/MS) analyses of sapogenins released after acid hydrolysis of the crude obtained saponin mixtures allowed to identify the aglycone moieties composing the different saponins. As shown in Table 2, the five extracts have different compositions in terms of dominant aglycones. Hederagenin and bayogenin are the main sapogenis in *M. heyniana* (37.6 ± 2.3% and 39.4 ± 0.8%, respectively) and *M. murex* (47.3 ± 0.9% and 36.9 ± 2.1%, respectively). The three aglycones hederagenin (23.2 ± 1.3%), bayogenin (19.0 ± 0.8%) and medicagenic acid (32.7 ± 1.3%) are dominant in *M. hybrida*, while *M. lupulina* is characterized by 46.4 ± 1.7% of medicagenic acid, 15.3 ± 0.2% of zanhic acid and 24.8% ± 0.6% of soyasapogenol B. This compound represents 51.6 ± 1.3% of the total in *M. truncatula,* with medicagenic acid (18.1 ± 0.8%) and zanhic acid (20.3 ± 0.7%) as the other two main aglycones.

Elucidation of the chemical structure of the saponins making up the five extracts was achieved based on their ESI-MS fragmentation and chromatographic behavior compared with authentic samples already fully identified by the authors in previous works and literature data [8,27,28,29,30,31,32]. Saponins were tentatively identified based on molecular ion [M-H]^−^, on key fragment ions and other MS observations. In general, the loss of 132 m/z was indicative of pentose (e.g., arabinose, xylose, apiose), the loss of 146 m/z was indicative of deoxyhexose (e.g., rhamnose), the loss of 162 m/z was indicative of hexose (e.g., glucose, galactose) and the loss of m/z 176 was indicative of hexuronic acid (e.g., glucuronic acid). The most abundant tentatively identified saponins in the five extracts are listed in Table 3.

High molecular weight compounds, in particular glycosides of medicagenic and zanhic acids, were detected in higher amounts in *M. hybrida*, *M. lupulina* and *M. truncatula* extracts. Shorter sugar chain saponins (2–3 sugars in the molecule) are instead most abundant in *M. heyniana* and *M. murex* extracts and represent a characteristic trait of these species. Soyasaponin I, a common saponin in the Fabaceae family, was detected in all the extracts in low amounts, but it represents more than 50% of the total saponins in *M. truncatula* extract.

### 2.2. Nematode Mortality Assay

The five saponin raw mixtures were poorly active on *M. incognita J2* at the 125 and 250 µg mL^−1^ concentrations, as nil or negligible mortality rates occurred for all the *Medicago* species (Table 4). At the 500 µg mL^−1^ concentration, *J2* mortality was above 90% after 16 h exposure to saponin extract of *M. hybrida* and *M. truncatula* or to 8 h contact with the saponin extract of *M. murex* s.

Adversely, the same concentration x exposure time combination occurred in 46.2% and 82.2% J2 mortalities for *M. heyniana* and *M. lupulina*, respectively. Mortality of *M. incognita J2* also ranged 90% after 8 h permanence in the 1000 µg mL^−1^ solutions of *M. heyniana* and *M. hybrida*. 

The 125 and 250 µg mL^−1^ solutions of saponin extract from *M. heyniana*, *M. hybrida* and *M. truncatula* poorly affected *X. index* females at all the exposure times, whereas a 26.8% mortality occurred after 8 h immersion in the 250 µg mL^−1^ solutions of *M. lupulina* and *M. murex* saponins (Table 5). The activity of M. *hybrida* and *M. truncatula* continued to be limited also at a 500 µg mL^−1^ concentration, as the mortaliy peaked only 23.3% at the longest exposure time. The same concentration resulted in 36.8% and 43.3% mortality after 16 h exposure to *M. heyniana* and *M. murex* saponins, respectively, or even in 93.3% mortality for the 8 h treatment with the *M. lupulina* saponin extract. 

An almost complete nematode mortality was recorded after 24 h treatment with the highest concentration of *M. heyniana*, *M. lupulina* and *M. truncatula* saponins, whereas the same concentration x time combination peaked 83.3 and 46.7% mortality rates for the saponins of *M. murex* and *M. hybrida*, respectively. Interestingly, the 1000 µg mL^−1^ concentration of M. lupulina caused total nematode mortality even after 4 h treatment.

The *J2* of *G. rostochiensis* was more sensitive to the five Medicago extracts than the other two nematode species. After a 4 h exposure, mortality rates ranged from 23% to 27% at the lowest concentration of all saponin mixtures, except for that of *M. truncatula*, and varied from 39 to 47% for the 1000 µg mL^−1^ solutions (Table 6). The higher sensitivity of *G. rostochiensis J2* was confirmed also at the longer exposure times, as after 24 h immmersion in 125 µg mL^−1^ solutions, the mortality rates ranged from about 56% of *M. truncatula* to 60–64% of M. murex and *M. heyniana*, respectively, up to more than 75% for *M. hybrida* and *M. lupulina*. As also found for *M. incognita* and *X. index*, the highest concentration of the five saponin mixtures generally resulted in *J2* mortality rates not significantly different from those of Oxamyl.

### 2.3. Egg Hatchability Bioassay

A one- or two-week permanence of *M. incognita* egg masses in all the saponin solutions of the five *Medicago* species significantly reduced the percentage egg hatch compared to the water control and, at the 1000 µg mL^−1^ concentration, also to the Oxamyl solution (Table 7). Egg hatch ranged from 52–47% to 39–18% at 125 µg mL^−1^ and 1000 µg mL^−1^, respectively, at the one-week exposure, and was furtherly reduced after a two-week treatment, ranging from 39–32% at 125 µg mL^−1^) to 21–10% 1000 µg mL^−1^. The hatchability of *G. rostochiensis* eggs was never reduced by one-week treatment of the cysts with any of the saponin solutions, as results were not significantly different from the 0.6 mM sodium metavanadate control for the 125-500 µg mL^−1^ solutions of *M. heyniana* saponin extract and the lowest concentration of *M. lupulina*, *M. murex* and *M. truncatula* extracts. 

Adversely, all the other one-week treatments with the saponin in solutions caused a significant increase of *G. rostochiensis* egg hatch, with an 80% peak for the 1000 µg mL^−1^ solutions of *M. hybrida* and *M. lupulina* saponins. This significant egg hatch increase was extended, according to a dose-related relationship, also to almost all the two-week treatments with the saponin solutions, with 93–96% percentage hatch peaks at the 1000 µg mL^−1^ concentrations of saponins from all the *Medicago* species except for *M. heyniana*. 

### 2.4. Experiment in Soil

Compared to the non-treated control, all the soil amendments with biomasses from the five *Medicago* species significantly suppressed *M. incognita* multiplication and gall formation on tomato roots as well as the final nematode population in soil (Table 8). Moreover, almost all the highest amendment rates were not significantly different, or even lower, than Oxamyl. 

Soil treatments with the *Medicago* plant materials always resulted in a significant dose-related increase of tomato growth compared to the non-treated soil and, at the highest rate, even to Oxamyl and the non-infested control (Table 8).

## 3. Discussion

The saponin extract of the five *Medicago* species was demonstrated as strongly active on *X. index* and, except for *M. hybrida*, on *M. incognita* at concentrations above 250 µg mL^−1^, as confirming the almost complete mortality previously reported for a 16 or 24 h exposure of *M incognita J2* and *X. index* females to a 500 μg mL^−1^ solution of *M. sativa* saponins [26]. As in this cited study, *G. rostochiensis J2* sensitivity to the five saponin mixtures was higher than that of *M. incognita* and *X. index* specimens, but lower than that previously showed to *M. sativa* saponins, which caused 40%–54% mortality even after a 4 and 8 h exposure, respectively, to a 125 μg mL^−1^ concentration [26]. Literature data on the activity of *Medicago* saponins on phytoparasitic nematodes are almost exclusively limited to the previous studies of our research group, which proved a biocidal activity of saponins, prosapogenins and sapogenins from *M. arborea*, *M. arabica* and *M. sativa* on *X. index* [25], as well as the activity of *M. sativa* saponin mixtures against the same nematode species tested in this study [26]. In addition, soil treatments with a crude extract of *M. sativa* saponins resulted in a significant reduction of *M. incognita* infestation on tomato [33]. More generally, only few data have been reported on the nematicidal effects of saponins from other plants. A significant effect on the motility of *M. incognita J2* was reported for the saponins from *Asparagus* spp. [34], and saponin solutions were found to significantly reduce the number of *M*. *javanica* eggs and viable *J2* both in vitro and in soil [13]. Furthermore, the total saponin fraction of *Portulaca oleracea* L. and *Lantana camara* L. strongly reduced the in vitro motility of *Meloidogyne* spp. *J2*, as well as almost completely inhibited gall formation on eggplant roots under greenhouse conditions [35]. Adversely, the single saponin fraction of a *Q. saponaria* extract showed a poor nematicidal effect on a range of phytoparasitic nematodes, including *X. index*, the northern root-knot nematode *M. hapla* Chitwood, *P. thornei*, *Tylenchorhynchus* sp. and *Helicotylenchus* sp. [14]. 

Crude saponin content of the five *Medicago* species under investigation, as ranging from 0.60% of *M. truncatula* to 1.62% of *M. heyniana*, is in good agreement with published data, which reported a crude saponin content of about 0.5%–1.5% dry matter in medics [27,36,37,38]. The high molecular weight compounds detected at higher amounts in *M. hybrida*, *M. lupulina* and *M. truncatula* extracts, in particular saponins containing glycosides of medicagenic and zanhic acids, were previously identified in the aerial parts of *Medicago* species [8], including *M. arborea* [28], *M. sativa* [31], *M. truncatula* [37] and *M. marina* [32]. Given that zanhic acid is synthesized in the green parts of the plants [39], its glycosides have not previously been found in *M. lupulina* and *M. hybrida* [8,38] when roots were investigated.

The biological activity of saponins is dependent on the number of side sugar chains attached to the sapogenins as well as to the nature of the sapogenin itself [8]. Thus, previous in vitro investigations allowed to relate the nematoxic effect of *M. sativa* saponins to the high amount of medicagenic acid. When pure aglycones have been used in in vitro bioassays, hederagenin was shown to be even more toxic than medicagenic acid and bayogenin against *X. index*, while soyasaponin I, containing soyasapogenol B as a glycone, was the less-active saponin [25]. This finding seems to be confirmed also in the present study by the nematotoxic properties against *X. index* displayed by the three species *M. heyniana*, *M. hybrida* and *M. murex,* which produce saponins with high amounts of the two aglycones hederagenin and bayogenin. In addition, *M. lupulina* with a high amount of medicagenic acid was particularly active against *M. incognita*.

The mechanisms of the nematicidal activity of saponins are still not clearly elucidated, as different hypotheses were suggested by literature studies. Changes in cell permeability following the specific interaction of saponins with cell membranes were generally hypothesized as a cause of biological effects of these compounds [8,25]. More recently, Ibrahim and Srour [33] observed a decrease of cholesterol in eggs of root-knot nematodes, according to a concentration effect relationship, following soil treatments with a *M. sativa* saponin extract.

As suggested in our previous studies [25], a specific interaction of saponins with the nematode cuticle ultrastructure may be also involved in their mechanism of activity. Based on this hypothesis, differences in the cuticle chemical components of the nematode species could account for the different response of the three nematode species tested in this study to the five saponin mixtures [26].

Nevertheless, we have already noticed in a previous study [5] that the nematicidal activity of plant extracts and/or pure metabolites may vary according to the nematode species and the life stage of the same nematode species. Thus, for example, we have already observed that the potato cyst nematode *G. rostiochiensis* was more susceptible than *M. incognita* to the toxic effect of *A. annua* and its active compound, artemisinin [5]. Consistently, *G. rostochiensis J2* was more sensitive to the five *Medicago* extracts than *M. incognita* and *X. index* also in the present study. 

The effect of the five saponin extracts on the hatchability of *M*. *incognita* and *G. rostochiensis* eggs was the exact opposite, as the percentage hatch of *M. incognita* eggs was significantly reduced and did not affect or even stimulate the hatchability of *G. rostochiensis* eggs. Effects of saponins on nematode egg hatchability were scarcely documented, as literature studies mainly described their effects on *J2* motility and viability or on nematode infestation on host plants. The toxicity of the five saponin solutions to *M. incognita* eggs agrees with their effects on infective *J2*, whereas the stimulation of *G. rostochiensis* egg hatchability can be considered an unexpected result. An increased permeability of the cyst wall by the interaction with saponins may be hypothesized, though the exact biochemical mechanisms need to be specifically investigated. 

The infestation of *M. incognita* on tomato plants was significantly reduced by soil amendments with the dry biomass of the five *Medicago* species, in full agreement with previous reports of a reduced infestation of *M. incognita* both on potted and field tomato or of the carrot cyst nematode *Heterodera carotae* Jones on field carrot following soil treatments with *M. sativa* biomass [40]. Moreover, previous studies also documented a significant suppression of phytoparasitic species *M. javanica*, *Paratrichodorus* sp. and *Criconemella xenoplax* joined to an increase of beneficial free-living nematodes, in a soil amended with *M. sativa* pelleted biomass [41], as well as an activity against a wide range of soilborne fungal strains of soil amendments with *M. truncatula* aerial parts [37]. 

Results from the in vitro experiments indicated that saponins from plant tissues of the five *Medicago* species are surely involved in the strong root-knot nematode suppression by soil incorporation of these plant materials. However, as already remarked in our previous studies [25,26], further contributory mechanisms should be also hypothesized, such as the nematoxicity of other bioactive metabolites from *Medicago* plants [42], an ammonia release by the decomposition of *Medicago* biomasses in soil [43,44] or an increase of phytonematode-suppressive microorganisms on the favorable substrate represented by *Medicago* tissues [45].

In particular it is known that *Medicago* species also synthesize polyphenolics such as glycosyl derivatives of apigenin, luteolin, chrysoeriol and tricin in *M. truncatula* [19,46] and in *M. sativa* [47,48]. Several biological functions related with plant structural protection, regulation of plant environmental communication and control of plant physiological events have been ascribed to plant polyphenolics. It has also been shown that they have a role as plant defense compounds against a range of microorganisms and that they are involved in plant–nematode interactions acting as defense compounds [3,49,50]. Thus, the presence of this type of metabolite in the biomass can reasonably be involved in the regulation of plant–nematode interactions when administered in soil to infested tomato plants.

The improved tomato growth in soil amended with the five *Medicago* species plant material also agrees with our previous reports of a biostimulating effect of soil incorporation with *M. sativa* biomass [42]. As previously noted for *M. sativa*, the growth effect of *Medicago* amendments may be related not only to the reduced nematode infestation but also to the physiological role of other specialized *Medicago* metabolites as well as to a general improvement of soil physical, chemical and microbiological properties such as an increased nitrogen content [51].

## 4. Materials and Methods 

### 4.1. Plant Material

The five *Medicago* species, namely *M. heyniana* Greuter, *M. hybrida* (Pourr.) Trautv, *M. lupulina* L., *M. murex* Willd and *M. truncatula* Gaertn, were grown at the Institute of Soil Science and Plant Cultivation, Poland, and harvested at the beginning of flowering stage. Leaves were lyophilized, finely powdered and stored in dry conditions until use.

### 4.2. Preparation of Saponin Extracts and Their Characterization

Saponin extracts were prepared according to a standard procedure [27,38]. Briefly, the powdered leaves from each *Medicago* species (about 200 g) were first defatted with CHCl_3_ in a Soxhlet apparatus and then saponins extracted with 80% MeOH under reflux (48 h). After removing the solvent with a rotary evaporator, the residue of each extract was resuspended in 30% MeOH and loaded onto a C18 column (Lichroprep RP-18, 50 × 20 mm, 40–63 μm, Merck, Darmstad, Germany), equilibrated with MilliQ water. Elution was performed with 40% MeOH (v/v) to remove some polar compounds, and saponins were then eluted with 80% MeOH (v/v). Purified samples were then dried under reduced pressure. Saponin mixtures, obtained as brownish powder, were kept in airtight vials until used. Three extractions were performed on each sample and results expressed as mean ± standard deviation (SD). The saponin mixtures were thus used for the successive analyses. Saponin extracts were checked by silica gel TLC plates, developed with ethyl acetate/acetic acid/water (7:2:2) and sprayed with the Liebermann–Burkhard reagent (MeOH/acetic anhydride/sulphuric acid, 10:1:1 v/v) to visualize the components.

#### 4.2.1. Hydrolysis of Saponins and Analysis of Sapogenins

Crude saponin mix (10–15 mg) was treated with 30 mL of 2N HCl in 50% aqueous MeOH under reflux for 8 h. After cooling, MeOH was eliminated under reduced pressure, 20 mL of water was added and then aglycones extracted with AcOEt (2 × 10 mL). Each of the organic solutions containing the aglycones was dried by rotary evaporation and used in the successive analyses. Three independent hydrolysis reactions were performed for each sample. 

Aglycone composition was determined by TLC, GC/FID and GC/MS methods by comparison to previously purified sapogenins from *Medicago* spp. [52]. Silica gel TLC was eluted with petroleum ether/CHCl_3_/AcOH (7:2:1), and spots were visualized by spraying the developed TLC with the Liebermann–Burkhard reagent followed by heating at 120 °C. Sapogenins were also identified by GC/FID and GC/MS as their methyl-silyl derivatives. Samples were dissolved in 0.5 mL of MeOH and treated with CH_2_N_2_. After solvent evaporation under a stream of nitrogen, silylation was performed by using 0.2 mL of a mixture of pyridine-hexamethyldisilazane-chlorotrimethylsilane (2:1:1) at 70 °C for 10 min. Reacted samples were properly diluted with isooctane and used for GC/FID and GC/MS analyses. Gas-chromatographic analyses were performed with a 30 m × 0.32 mm, 0.25 μm i.d., DB-5 capillary column as described in Tava et al. [52]. Retention times and mass spectra data were compared to those of previously purified and identified sapogenins. The relative amount of each sapogenin was calculated as peak area percent relative to total peak area from GC/FID analysis of the whole saponin extract. For each *Medicago* species under investigation, results were expressed as the mean of three independent evaluations ± SD.

#### 4.2.2. LC/MS Analyses

The investigated extracts were also subjected to UHPLC-ESI-MS analyses, using an ACQUITY UPLC chromatographic system (Waters, Milford, MA, USA), coupled with a PDA detector and a triple quadrupole mass detector (ACQUITY TQD, Waters). Saponins were separated on an ACQUITY HSS C18 column (2.1 × 100 mm, 1.8 µm; Waters), maintained at 40 °C; the injection volume was 2.5 µL. The elution method was: 0.0–0.5 min, 1% of solvent B (acetonitrile with 0.1% formic acid) in solvent A (0.1% formic acid in MilliQ water); 0.5–25.5 min, a linear gradient to 50% B. The column was then washed with 99% B (2 min) and re-equilibrated with 1% B (1.95 min) to return to the initial gradient. The flow rate was 0.400 mL min^−1^. The mass detector operated in negative ion mode. Capillary voltage was 3.0 kV; cone voltage was 40 V; source temperature was 140 °C; desolvation temperature was 350 °C; desolvation gas flow was 800 L h^−1^; cone gas flow was 100 L h^−1^. Full-scan spectra were acquired in the range from 150 to 1600 m/z.

### 4.3. Nematode Mortality Bioassays

An Italian population of *M. incognita* was preliminarily multiplied on plants of tomato cv. Roma in a glasshouse maintained at 25 ± 2 °C constant temperature. Formed egg masses were collected from the infested roots and incubated in distilled water in a growth chamber at 25 °C. An Italian population of *G. rostochiensis* Ro1 was reared on potato cv. Spunta in a glasshouse at 20 °C. Nematode cysts were extracted from the soil by Fenwick’s flotation technique [53] and then incubated at 20 °C in a 0.6 mM sodium metavanadate hatching solution [54]. The emerged *J2* of both species were collected and stored in water at 5 °C until their use. The adult females of *X. index* were extracted from the soil of an infested vineyard located at Ginosa (Taranto province) by the Cobb’s decanting and sieving method [55] and immediately used. All the three nematode species were preliminarily identified by a morphological characterization under an optical microscope. About 150 *J2* of *M. incognita* or *G. rostochiensis* and 20 adult females of *X. index* were suspended in 0.5 mL distilled water in 1.5 mL Eppendorf vials. A 0.5 mL volume of 2000, 1000, 500 and 250 μg mL^−1^ aqueous solutions of the saponin extract of *M. heyniana*, *M. truncatula*, *M. murex*, *M. hybrida* and *M. lupulina* was added to each vial to obtain final concentrations of 1000, 500, 250 and 125 μL mL^−1^. Nematodes were exposed to each concentration of the five saponin solutions for 4, 8, 16 or 24 h, during which the Eppendorf tubes were maintained in agitation. There were four replicates for each concentration x exposure time combination, and vials were arranged in a completely randomized experimental design. Distilled water and a 1 mL L^−1^ water solution of a liquid formulation (10% a.i.) of the nematicide Oxamyl were included as controls.

At the end of each exposure time, the nematodes from each replicate of each treatment were observed under a light microscope, assuming the complete immobility of *M. incognita* and *G. rostochiensis J2* and of needle-pricked *X. index* females [25] as evidence of the solution toxicity. The observed specimens were recovered on a 5 μm sieve, repeatedly washed with water and then transferred to distilled water. Nematodes were considered dead if their immobility persisted after a 72 h permanence in water. Mortality rates were calculated according to Abbott’s formula [55] m = 100 × (1-nt/nc), in which m = percent mortality; nt = number of viable nematodes after the treatment; nc = number of viable nematodes in water. Two experimental runs each with a separate control were carried out on each nematode species. 

### 4.4. Egg Hatchability Bioassays

Groups of 50 *M. incognita* egg masses or *G. rostochiensis* cysts, averaging 400 eggs per mass and 700 eggs per cyst, respectively, were placed in 2 cm diameter sieves (215 μm aperture) and submerged with 3 mL of the 500 and 1000 μL ml^−1^ solutions of the five saponin extracts within a 3.5 cm diameter Petri dish. The experiments on *G. rostochiensis* cysts were prepared in a 0.6 mM water solution of sodium metavanadate, as reported as a hatching agent for this nematode species [54]. The egg masses and the cysts were exposed to each test solution for 1 or 2 weeks in a growth chamber at 25 and 20 °C, respectively. As in the first experiment, four replicates were provided for each treatment in comparison, arranging the experimental units in a complete randomized block design. Distilled water and pure 0.6 mM sodium metavanadate solution were used as controls for egg masses and cysts, respectively, whereas the treatment with the same 1 mL L^−1^ water solution of Oxamyl solution used in the mortality assay was included as a chemical control. At the end of each exposure time, egg masses and cysts were removed from the test solutions and the hatching test continued in distilled water or in the 0.6 mM sodium metavanadate solution, respectively. The emerged juveniles were removed and counted at weekly intervals. After each weekly removal of the emerged *J2*, egg masses and cysts were checked under a microscope, as to verify the occurrence of microbial contamination, and repeatedly washed with sterile water before renewing distilled water or 0.6 mM sodium metavanadate solution. All these weekly operations were carried out under a laminar flow cabinet, as to avoid microbial contaminations potentially affecting the egg hatch. The *M. incognita* egg masses were removed from the sieves after a total of five weeks and then dissolved by a 3 min shaking in a 1% sodium hypochlorite aqueous solution [56]. The hatching test on *G. rostochiensis* was prolonged for eight weeks, after which cysts were crushed according to the Bijloo’s modified method [57]. The unhatched eggs of both species were counted under a stereoscope. Egg hatchability was expressed as percentage ratio of total emerged *J2* to the total egg content of egg masses or cysts. Both experiments were repeated twice with separate controls for each experiment.

### 4.5. Experiment in Soil 

Roots of tomato cv Roma infested by the same population of *M. incognita* used for the experiments in vitro were minutely minced and thoroughly mixed. The number of eggs and *J2* per gram of roots was determined by processing six 10 g samples by the Hussey and Barker’s method [56] and then counting the extracted eggs and *J2* under a microscope. A steam sterilised sandy soil (64.4% sand, 18.7% silt, 16.9% clay, 0.8% organic matter and 7.5 pH) was added with appropriate amounts of the above infested roots, as to reach 8 eggs and *J2* mL^−1^ initial nematode population density. Dry green biomass of the five *Medicago* species was thoroughly mixed to the infested soil at 10, 20 or 40 g kg^−1^ soil rates, and mixtures were poured into 1.2 L clay pots. Non-treated soil, either non-infested or infested by *M. incognita*, and soil treated with the liquid formulation of Oxamyl (10% a.i.) used in the in vitro experiments, applied at an amount corresponding to a 2 L ha^−1^ a.i. field rate three days before transplanting, were included as controls. Pots were arranged on greenhouse benches according to a randomized block design with five replicates of each treatment in comparison.

One tomato cv Tomito seedling (1 month old) was transplanted in each pot two weeks after soil amendments with the *Medicago* plant material. Tomato plants were maintained at a constant temperature of 25 ± 2 °C throughout a two-month period, at the end of which fresh weight of aerial parts and roots was recorded on each replicate. Root gall infestation was estimated on each tomato plant according to the Taylor and Sasser’s scale (0 = no galls, 1 = 1–2 galls, 2 = 3–10 galls, 3 = 11–30 galls, 4 = 31–100 galls and 5 > 100 galls) [58]. Final nematode population density was determined by extracting nematode eggs and *J2* from a 10 g sample of each tomato root [58] and from a 500 mL sample of soil from each pot [59].

### 4.6. Statistical Analysis 

The arcsin transformed pooled data from the two experimental runs of the in vitro experiments and the Ln (x + 1) transformed nematode data and raw plant growth data from the experiment in soil were subjected to one-way analysis of variance, comparing means by the Least Significant Difference Test at *p* ≤ 0.05 [57]. The LC50 values of each saponin extract were also calculated with data from both in vitro assays by a probit-logistic analysis [55]. PlotIT 3.2 (Scientific Programming Enterprises, Haslett, MI, USA) software was used to perform all the stastistical analyses.

## 5. Conclusions

Results from this study demonstrate that saponin-rich extracts and plant biomasses from *M. heyniana*, *M. hybrida*, *M. lupulina*, *M. murex* and *M. truncatula* can be highly suppressive to root-knot nematodes and, therefore, could be included among the potential sources of new sustainable nematicidal products addressed to a management of phytoparasitic nematodes, also in a synergistic combination with other bio-derived products or other nonchemical techniques.

The presence on the market of products derived from saponin-rich extracts from *Q. saponaria* seems to demonstrate that an industrial exploitation of the five studied *Medicago* species may be technically and economically feasible, also due to the large biomass produced by these plants. Analogously, the already available nematicidal products based on dry biomasses of biofumigating Brassicaceae plants indicate potential granular or powdered formulations of the biomasses from the five *Medicago* species as a reasonable alternative to saponin extract-based products, also in consideration of the high suppressiveness demonstrated by soil amendments with the *Medicago* biomasses.

As generally remarked for plant-derived pesticides, an extended evaluation of the impact of potential *Medicago* plant-based products on other biotic soil components should be preliminarily undertaken, and agronomical techniques and plant-growing conditions should be preliminarily set up, as to avoid unstable effects on target nematodes related to a variable content of saponins and other bioactive compounds. 

## Figures and Tables

**Table 1:** Saponin content (% dry matter ± SD) in leaves of the five *Medicago* spp.

*Medicago* spp.	Saponin Content
*M. heyniana*	1.62 ± 0.37
*M. hybrida*	0.64 ± 0.03
*M. lupulina*	0.44 ± 0.04
*M. murex*	1.30 ± 0.05
*M. truncatula*	0.60 ± 0.05

**Table 2:** Sapogenin composition (expressed as % ± SD of total sapogenins) obtained by acid hydrolysis of saponins extracted by the five *Medicago* spp.

Sapogenin	*M. heyniana*	*M. hybrida*	*M. lupulina*	*M. murex*	*M. truncatula*
Oleanolic acid	4.2 ± 1.3	5.9 ± 1.2	-	2.8 ± 0.3	-
Hederagenin	37.6 ± 2.3	23.2 ± 1.3	<0.1	43.7 ± 0.9	-
Bayogenin	39.4 ± 0.8	19.0 ± 0.8	2.6 ± 0.1	36.9 ± 2.1	0.8 ± 0.1
Medicagenic acid	2.8 ± 0.7	32.7 ± 1.3	46.4 ± 1.7	1.7 ± 0.3	18.1 ± 0.8
Zanhic acid	3.8 ± 0.1	13.6 ± 0.7	15.3 ± 0.2	0.7 ± 0.2	20.3 ± 0.7
Soyasapogenol A	0.9 ± 0.3	-	6.0 ± 0.4	1.0 ± 0.2	3.7 ± 0.4
Soyasapogenol B	6.4 ± 1.7	4.3 ± 0.3	24.8 ± 0.6	7.4 ± 0.5	51.6 ± 1.3

**Table 3:** The most abundant tentatively identified saponins in the five *Medicago* spp. and their quantitative evaluation (% of total saponins).

	Rt	[M-H]^−^	MS fragments	Tentative identification	%
*M. heyniana*	17.32	825	501[M-H-162-162]^−^(Med)	Med-Hex-Hex	2.6
	17.80	795	663[M-H-162]^−^; 471[M-H-162-162]^−^(Hed)	Hed-Hex-Hex	1.6
	19.14	765	603[M-H-162]^−^; 471[M-H-162-132]^−^(Hed)	Hed-Pen-Hex	20.3
	19.26	765	633[M-H-132]^−^; 471[M-H-132-162]^−^(Hed)	Hed-Pen-Hex	6.8
	20.08	811	767[M-H-CO_2_-H_2_O]^−^; 487[M-H-162-162]^−^(Bayo)	Bayo-Hex-Hex	16.4
	21.39	811	649[M-H-162]^−^; 487[M-H-162-162]^−^(Bayo)	Bayo-Hex-Hex	5.5
	22.06	941	879[M-H-CO_2_-H_2_O]^−^;795[M-H-146]^−^;633[M-H-146-162]^−^; 457[M-H-146-132-176]^−^(SoyaB)	Soya B-HexA-Hex-dHex (SSI)	5.2
	23.62	765	603[M-H-162]^−^; 471[M-H-132-162]^−^(Hed)	Hed-Pen-Hex	7.5
*M. hybrida*	15.14	1397	1265[M-H-132]^−^; 1011[M-H-CO_2_-H_2_O-162-162]^−^; 455[M-H-CO_2_-H_2_O-162-162-146-146-132-132]^−^(Zanh-CO_2_-H_2_O)	Zanh-Hex-Hex-dHex-dHex-Pen-Pen	24.1
	15.98	1235	1103[M-H-132]^−^; 1011[M-H-CO_2_-H_2_O-162]^−^; 455[M-H-CO_2_-H_2_O-162-146-146-132-132]^−^(Zanh-CO_2_-H_2_O)	Zanh-Hex-dHex-dHex-Pen-Pen	3.6
	16.90	825	663[M-H-162]^−^; 601[M-H-162-CO_2_-H_2_O]^−^; 487[M-H-162-176]^−^(Bayo)	Bayo-HexA-Hex	7.0
	17.19	971	809[M-H-162]^−^; 633[M-H-162-176]^−^; 471[M-H-162-176-162]^−^(Hed)	Hed-HexA-Hex-Hex	2.0
	17.51	825	663[M-H-162]^−^; 501[M-H-162-162]^−^(Med)	Med-Hex-Hex	26.5
	17.83	795	663[M-H-162]^−^; 471[M-H-162-162]^−^(Hed)	Hed-Hex-Hex	3.6
	21.39	811	649[M-H-162]^−^; 487[M-H-162-162]^−^(Bayo)	Bayo-Hex-Hex	4.3
	22.05	941	879[M-H-CO_2_-H_2_O]^−^;795[M-H-146]^−^; 633[M-H-146-162]^−^; 457[M-H-146-132-176]^−^(SoyaB)	Soya B-HexA-Hex-dHex (SSI)	1.2
	22.56	795	633[M-H-162]^−^; 471[M-H-162-162]^−^(Hed)	Hed-Hex-Hex	4.0
*M. lupulina*	15.90	1103	927[M-H-176]^−^; 909[M-H-176-H_2_O]^−^; 517[M-H-176-410]^−^(Zanh)	Zanh-HexA-dHex-Pen-Pen	6.1
	17.63	1219	1043[M-H-176]^−^; 911[M-H-176-132]^−^; 501[M-H-176-132-410]^−^(Med)	Med-HexA-dHex-Pen-Pen-Pen	9.2
	17.87	1087	911[M-H-176]^−^; 501[M-H-176-410]^−^(Medic)	Med-HexA-dHex-Pen-Pen	45.8
	21.96	911	765[M-H-146]^−^; 457[M-H-146-132-176]^−^(SoyaB)	Soya B-HexA-Pen-dHex	13.0
	22.05	941	879[M-H-CO_2_-H_2_O]^−^;795[M-H-146]^−^;633[M-H-146-162]^−^; 457[M-H-146-132-176]^−^(SoyaB)	Soya B-HexA-Hex-dHex (SSI)	7.2
*M. murex*	17.10	971	809[M-H-162]^−^; 663[M-H-162-146]^−^; 487[M-H-162-146-176]^−^(Bayo)	Bayo-HexA-dHex-Hex	3.2
	18.92	955	793[M-H-162]^−^; 647[M-H-162-146]^−^; 471[M-H-162-146-176]^−^(Hed)	Hed-HexA-Pen-Hex	7.7
	20.09	811	767[M-H-CO_2_-H_2_O]^−^; 487[M-H-162-162]^−^(Bayo)	Bayo-Hex-Hex	44.1
	22.03	941	879[M-H-CO_2_-H_2_O]^−^;795[M-H-146]^−^;633[M-H-146-162]^−^; 457[M-H-146-132-176]^−^(SoyaB)	Soya B-HexA-Hex-dHex (SSI)	28.7
	22.56	795	633[M-H-162]^−^; 471[M-H-162-162]^−^(Hed)	Hed-Hex-Hex	1.5
	22.85	765	619[M-H-146]^−^; 487[M-H-146-132]^−^(Bayo)	Bayo-dHex-Pen	5.3
	23.64	765	603[M-H-162]^−^; 471[M-H-132-162]^−^(Hed)	Hed-Hex-Pen	4.0
*M. truncatula*	15.70	1383	997[M-H-162-162-CO2-H2O]^−^; 841[M-H-132-410]^−^; 455[M-H-CO_2_-H_2_O-132-410-162-162]^−^(Zanh-CO_2_-H_2_O)	Zanh-Hex-Hex-Pen-dHex-Pen-Pen	3.1
	15.82	1383	997[M-H-162-162-CO2-H2O]^−^; 841[M-H-132-410]^−^; 455[M-H-CO_2_-H_2_O-132-410-162-162]^−^(Zanh-CO_2_-H_2_O)	Zanh-Hex-Hex-Pen-dHex-Pen-Pen	11.9
	17.56	1367	1235[M-H-132]^−^; 981[M-H-162-162-CO_2_-H_2_O]^−^; 849[M-H-162-162-CO_2_-H_2_O-132]^−^; 439(Med-CO_2_-H_2_O)	Med-Hex-Hex-Pen-dHex-Pen-Pen	8.9
	17.87	1087	911[M-H-176]^−^; 677[M-H-410]^−^; 501[M-H-176-410]^−^(Med)	Med-HexA-Pen-dHex-Pen	2.3
	21.96	911	765[M-H-146]^−^; 457[M-H-146-132-176]^−^(SoyaB)	Soya B-HexA-Pen-dHex	9.0
	22.05	941	879[M-H-CO_2_-H_2_O]^−^;795[M-H-146]^−^;633[M-H-146-162]^−^; 457[M-H-146-132-176]^−^(SoyaB)	Soya B-HexA-Hex-dHex (SSI)	54.2

**Table 4:** Mortality (%) of *M. incognita J2* after 4 to 24 h exposures to 125–1000 µg mL^−1^ solutions of the saponin extract from five different *Medicago* species (means ± SE).

Concentration	Exposure Time (hours)			
(µg mL^−1^)	4	8	16	24
	***M. heyniana***			
125	0.8 ± 0.2	0.9 ± 0.2	46.4 ± 1.7	1.7 ± 0.3
250	1.4 ± 0.4	1.5 ± 0.6	2.3 ± 0.4	2.0 ± 0.6
500	1.5 ± 0.5	3.3 ± 0.9	46.0 ± 3.9	90.1 ± 0.4
1000	1.3 ± 0.3	89.8 ± 3.0	91.1 ± 2.3	97.9 ± 0.3
LC50	≥	798	521	353
	***M. hybrida***			
125	1.6 ± 0.4	2.8 ± 0.3	3.4 ± 0.4	3.4 ± 0.2
250	2.4 ± 1.0	3.4 ± 0.2	3.6 ± 0.2	3.7 ± 0.2
500	2.4 ± 0.6	23.6 ± 2.1	95.9 ± 1.0	95.1 ± 0.2
1000	3.4 ± 0.9	88.1 ± 3.0	96.8 ± 0.5	95.8 ± 0.4
LC50	≥	612	353	363
	***M. lupulina***			
125	0.5 ± 0.2	2.7 ± 0.4	3.3 ± 0.4	3.5 ± 0.2
250	0.5 ± 0.3	3.7 ± 0.5	5.8 ± 0.6	6.1 ± 0.4
500	4.3 ± 0.5	5.2 ± 0.4	82.2 ± 1.7	89.2 ± 0.3
1000	7.8 ± 1.0	11.5 ± 2.0	89.3 ± 3.1	90.2 ± 1.8
LC50	≥	≥	422	404
	***M. murex***			
125	1.4 ± 0.3	1.9 ± 0.7	1.9 ± 0.3	2.1 ± 0.5
250	1.5 ± 0.4	2.0 ± 0.6	2.2 ± 0.7	2.8 ± 0.4
500	2.0 ± 0.6	91.3 ± 3.6	91.4 ± 3.0	92.1 ± 0.2
1000	2.7 ± 1.0	92.3 ± 1.4	96.0 ± 0.5	95.5 ± 0.6
LC50	≥	414	383	385
	***M. truncatula***			
125	0.9 ± 0.2	1.6 ± 0.4	2.0 ± 1.5	4.4 ± 0.4
250	1.1 ± 0.1	4.7 ± 0.3	4.8 ± 0.1	4.7 ± 0.2
500	1.2 ± 0.2	33.3 ± 3.8	90.4 ± 1.8	90.6 ± 0.3
1000	2.7 ± 1.1	38.3 ± 1.3	91.4 ± 4.4	95.5 ± 1.8
LC50	≥	1098	416	360
Oxamyl (1 mL L^−^^1^)	2.8 ± 0.3	40.0 ± 1.0	88.2 ± 0.5	94.5 ± 0.7
Water	0.0	0.0	0.0	0.0
LSD 0.05	1.7	4.7	5.0	1.9

**Table 5:** Mortality (%) of *X. index* adult females after 4 to 24 h exposures to 125–1000 µg mL^−1^ solutions of the saponin extract from five different *Medicago* species (means ± SE).

Concentration	Exposure Time (hours)			
(µg mL^−1^)	4	8	16	24
	***M. heyniana***			
125	0.0	0.0	0.0	3.3 ± 2.4
250	0.0	0.0	0.0	3.3 ± 0.6
500	0.0	6.8 ± 4.7	36.8 ± 4.1	56.7 ± 6.2
1000	40.0 ± 4.1	66.8 ± 10.3	80.0 ± 6.2	100
LC50	≥	856	611	544
	***M. hybrida***			
125	0.0	0.0	0.0	3.3 ± 2.4
250	0.0	3.3 ± 2.4	3.3 ± 2.4	6.7 ± 2.4
500	0.0	6.8 ± 2.4	13.3 ± 2.4	23.3 ± 2.2
1000	3.2 ± 2.4	6.8 ± 4.7	30.0 ± 4.1	46.7 ± 2.8
LC50	≥	≥	1692	1165
	***M. lupulina***			
125	6.8 ± 2.4	6.8 ± 2.4	12.3 ± 1.6	6.7 ± 2.4
250	20.0 ± 8.2	26.8 ± 2.4	33.3 + 2.4	43.3 ± 4.7
500	56.7 ± 16.5	93.3 ± 2.4	96.7 ± 2.5	100
1000	100 ± 0	100	100	100
LC50	457	274	241	273
	***M. murex***			
125	0.0	13.3 ± 2.4	26.7 ± 2.4	36.7 ± 4.7
250	0.0	26.8 ± 6.2	40.0 ± 4.7	43.3 ± 6.2
500	0.0	33.3 ± 2.4	43.3 ± 2.4	50.0 ± 4.1
1000	16.8 ± 4.7	43.3 ± 2.4	63.3 ± 4.1	83.3 ± 2.4
LC50	≥	1357	536	295
	***M. truncatula***			
125	0.0	0.0	0.0	0.0
250	6.8 ± 2.4	6.8 ±2.4	6.7 ± 2.3	10.0 ± 1.1
500	13.3 ± 4.7	16.8 ± 4.7	23.3 ± 3.3	23.3 ± 2.4
1000	30.0 ± 7.1	86.8 ± 4.7	96.7 ± 2.4	97.6 ± 3.2
LC50	2228	628	525	498
Oxamyl (1 mL L^−^^1^)	38.5 ± 4.1	80.0 ± 4.1	95.0 ± 2.9	100
Water	0.0	0.0	0.0	0.0
LSD 0.05	14.5	14.3	15.6	18.4

**Table 6:** Mortality (%) of *G. rostochiensis J2* after 4 to 24 h exposures to 125–1000 µg mL^−1^ solutions of the saponin extract from five different *Medicago* species (means ± SE).

Concentration	Exposure Time (hours)			
(µg mL^−1^)	4	8	16	24
	***M. heyniana***			
125	22.7 ± 1.7	39.5 ± 1.3	42.7 ± 0.6	64.5 ± 1.4
250	29.6 ± 3.2	43.0 ± 2.3	47.9 ± 1.0	72.1 ± 3.0
500	37.7 ± 6.0	48.5 ± 4.0	67.8 ± 4.0	80.1 ± 1.7
1000	38.9 ± 5.6	55.8 ± 4.6	80.8 ± 3.9	88.2 ± 1.4
LC50	2648	546	212	52
	***M. hybrida***			
125	25.8 ± 1.5	29.0 ± 1.3	62.9 ± 4.4	76.3 ± 4.5
250	28.7 ± 2.9	32.2 ± 2.6	64.0 ± 4.0	77.0 ± 1.2
500	33.6 ± 1.3	37.5 ± 1.0	65.0 ± 5.6	87.0 ± 4.0
1000	44.7 ± 4.0	47.9 ± 3.8	82.6 ± 4.6	87.8 ± 1.5
LC50	2191	1538	54	9
	***M. lupulina***			
125	23.1 ± 0.6	27.4 ± 2.2	73.0 ± 4.8	75.5 ± 4.7
250	29.3 ± 1.9	30.0 ± 1.5	79.3 ± 4.4	79.8 ± 1.8
500	33.4 ± 3.7	41.9 ± 2.7	85.6 ± 4.8	85.4 ± 3.2
1000	40.7 ± 2.7	47.8 ± 1.4	86.9 ± 1.8	86.7 ± 1.9
LC50	2868	1215	10	5
	***M. murex***			
125	26.6 ± 0.9	29.3 ± 2.8	34.5 ± 2.4	60.1 ± 4.8
250	31.1 ± 2.1	34.1 ± 1.6	47.1 ± 1.1	61.8 ± 2.7
500	35.4 ± 2.0	44.9 ± 0.7	57.1 ± 0.9	72.6 ± 2.4
1000	46.8 ± 1.8	54.5 ± 4.2	72.7 ± 1.1	84.9 ± 2.6
LC50	1688	751	302	84
	***M. truncatula***			
125	10.8 ± 3.4	24.9 ± 1.3	33.4 ± 1.0	55.8 ± 5.0
250	22.7 ± 2.1	30.0 ± 2.1	52.6 ± 1.6	62.4 ± 2.0
500	25.7 ± 3.3	43.0 ± 4.2	67.8 ± 0.9	75.1 ± 1.2
1000	40.8 ± 3.4	51.7 ± 4.1	76.6 ± 2.1	85.6 ± 2.3
LC50	1755	895	245	104
Oxamyl (1 mL L^−^^1^)	34.5 ± 1.7	61.7 ± 1.3	78.3 ± 0.5	88.0 ± 0.7
Water	0.0 ±	0.0	0.0	0.0
LSD 0.05	8.3	6.6	12.9	8.0

**Table 7:** Hatchability (%) of *M. incognita* and *G. rostochiensis* eggs after a 1- or 2-week exposure of egg masses and cysts, respectively, to 125–1000 µg mL^−1^ solutions of the saponin extract from five different *Medicago* species (means ± SE).

Concentration	1 week		2 weeks	
(µg mL^−1^)	*M. incognita*	*G. rostochiensis*	*M. incognita*	*G. rostochiensis*
	***M. heyniana***			
125	47.0 ± 2.1	33.0 ± 0.6	38.7 ± 2.2	35.7 ± 1.2
250	33.0 ± 1.0	39.3 ± 1.2	26.0 ± 1.5	43.3 ± 0.9
500	24.3 ± 0.9	42.0 ± 2.1	17.7 ± 1.2	48.0 ± 2.1
1000	24.0 ± 1.1	46.7 ± 3.2	15.0 ± 0.6	69.3 ± 2.2
	***M. hybrida***			
125	49.0 ± 1.5	44.0 ± 1.5	32.0 ± 1.2	51.0 ± 2.5
250	39.7 ± 2.3	59.0 ± 1.0	23.3 ± 0.9	61.7 ± 1.8
500	28.7 ± 1.4	71.7 ± 0.7	18.7 ± 0.7	85.0 ± 1.7
1000	18.3 ± 0.9	80.0 ± 9.3	16.0 ± 0.6	95.7 ± 1.3
	***M. lupulina***			
125	51.7 ± 1.4	36.3 ± 0.3	42.3 ± 0.7	37.3 ± 0.3
250	46.7 ± 0.3	47.3 ± 0.7	38.0 ± 1.0	49.0 ± 0.0
500	34.7 ± 0.3	55.7 ± 0.9	26.7 ± 0.7	60.7 ± 3.5
1000	20.3 ± 0.9	80.7 ± 0.9	10.0 ± 0.6	93.3 ± 2.0
	***M. murex***			
125	51.0 ± 1.0	38.3 ± 0.9	35.3 ± 1.8	45.3 ± 2.0
250	41.7 ± 1.2	53.0 ± 2.3	28.7 ± 0.9	61.3 ± 0.9
500	33.7 ± 0.9	60.0 ± 2.0	23.3 ± 0.7	72.3 ± 0.3
1000	21.7 ± 0.7	67.7 ± 1.9	19.0 ± 1.0	93.3 ± 3.3
	***M. truncatula***			
125	52.0 ± 2.1	39.3 ± 1.8	39.0 ± 0.6	45.3 ± 1.4
250	45.3 ± 0.3	46.7 ± 2.2	34.0 ± 1.2	55.7 ± 1.8
500	39.7 ± 1.5	52.0 ± 4.5	23.0 ± 1.5	62.7 ± 0.9
1000	39.0 ±1.2	71.0 ± 2.1	21.3 ± 0.7	96.7 ± 1.2
Oxamyl (1 mL L^−^^1^)	29.3 ± 1.2	25.3 ± 0.9	20.0 ± 1.2	15.7 ± 0.9
Control	56.3 ± 0.7	35.7 ± 1.8	56.3 ± 0.7	35.7 ± 1.8
LSD 0.05	3.6	7.6	3.1	5.1

**Table 8:** Effect of soil amendments with dry plant biomass of the five tested *Medicago* species on the infestation of the root-knot nematode *M. incognita* and on the growth of tomato cv. Regina (means ± SE).

Amendment Rate	Nematode Eggs and *J2*		Root Gall	Plant Fresh Weight (g)	
(g kg^−1^ soil)	g^−1^ roots (x 1000)	(mL^−1^ soil)	Index (0-5)	Aerial Parts	Roots
		***M. heyniana***			
10	5.2 ± 0.2	14.0 ± 0.5	4.4 ± 0.2	32.4 ± 1.3	8.8 ± 0.2
20	3.5 ± 0.1	6.8 ± 0.4	4.0 ± 0.3	43.0 ± 0.7	12.8 ± 0.5
40	1.5 ± 0.1	4.4 ± 0.4	2.2 ± 0.4	51.4 ± 1.0	20.4 ± 0.6
		***M. hybrida***			
10	5.1 ± 0.4	13.6 ± 0.2	4.4 ± 0.4	30.2 ± 1.6	5.6 ± 0.4
20	3.6 ± 0.2	6.2 ± 0.5	3.8 ± 0.2	38.4 ± 0.8	10.8 ± 0.7
40	1.6 ± 0.3	4.0 ± 0.3	3.2 ± 0.2	47.4 ± 2.1	15.8 ± 0.4
		***M. lupulina***			
10	7.0 ± 0.3	7.2 ± 0.4	4.6 ± 0.2	33.2 ± 1.1	9.0 ± 0.3
20	2.8 ± 0.4	6.4 ± 0.5	3.6 ± 0.2	42.6 ± 1.4	11.6 ± 0.2
40	0.9 ± 0. 01	5.0 ± 0.3	2.4 ± 0.3	56.6 ± 1.2	19.2 ± 1.4
		***M. murex***			
10	5.4 ± 0.3	10.0 ± 0.5	3.8 ± 0.4	35.4 ± 0.8	5.0 ± 0.3
20	3.1 ± 0.2	4.0 ± 0.5	2.8 ± 0.5	43.6 ± 1.2	7.4 ± 0.2
40	1.5 ± 0.1	1.8 ± 0.2	2.2 ± 0.4	60.8 ± 1.5	14.4 ± 0.8
		***M. truncatula***			
10	7.1 ± 0.3	14.2 ± 0.4	4.6 ± 0.2	33.2 ± 1.2	5.6 ± 0.4
20	4.6 ± 0.4	6.8 ± 0.2	3.2 ± 0.4	45.4 ± 0.9	11.2 ± 0.4
40	1.7 ± 0.2	2.4 ± 0.2	2.8 ± 0.4	60.6 ± 0.9	20.2 ± 0.6
Oxamyl (2 L ha^−^^1^)	1.9 ± 0.2	5.2 ± 0.4	1.8 ± 0.2	42.0 ± 0.7	9.2 ± 0.2
Non treated	11.7 ± 0.7	21.4 ± 0.7	4.8 ±0.2	16.8 ± 0.7	2.6 ± 0.2
Non infested	-		-	52.2 ± 1.8	6.0 ± 1.5
LSD 0.05	0.9	1.1	0.9	3.4	1.8
